# A Physician to Physicians

**Published:** 1894-06-30

**Authors:** 


### The Hospital

An Institutional Journal of

The Medical Sciences and Hospital Administration, with

AN EXTRA NURSING SUPPLEMENT.

Vol. XVI.—No. 405.] [June 30, 1894.

### A Physician to Physicians.

The American Dr. Jacobi, whose work as a physician, and more than a physician, for children is well-known in two hemispheres, addressed a remonstrance to the medical profession at the recent Roman Medical Congress which is pathetic in its almost painful earnestness. *Non nocere* was his text, "See that you do not harm your patients." He would be a bold man who should say Dr. Jacobi's sermon was unnecessary. It is not every medical man who makes it a primay part of his business to conserve his patients' safety. This is hardly to be wondered at. The highest type of the physician, like the poet, is born and not made. In these times the profession has small regard for the born physician; it prefers the article which is obviously manufactured; and the more manifest are the marks of the factory of origin the more is the finished article applauded by the profession. That is a state of the medical mind which tends to nothing so much in practice as to a uniform mediocrity.

The tone of the profession, Dr. Jacobi thinks, is not high where it should be high. The modern medical man is too often a mere "axe grinder." He does not pursue medicine because he loves the science or loves his patients, but because he loves money and an easy life. It is not the ordinary family practitioner who is thus held up to reprobation by Dr. Jacobi, but the man who at the present moment is the most popular and sought after both inside the profession and outside: it is the specialist. It will be universally conceded that Dr. Jacobi himself is a specialist, and a very eminent one. Yet of a certain class of specialists he speaks with unsparing disapproval. This is well worthy of consideration from the standpoint of the actual progress and improvement of medical science and art. Is specialism, as now carried out, conducive to the scientific culture, practical capacity, and moral elevation of the specialist? Dr. Jacobi, without hesitation, answers this threefold question in the negative. "A young medical man," to use his own words, "who runs off into a specialty, honestly believing that a human organ can be studied and treated separately, like the wheel of a watch, has not intellect enough to be a physician, and ought to have been discouraged from entering the ranks." Many of us in this country have long known such a judgment to be sound: we are glad that a veteran like Dr. Jacobi has had the courage to give the judgment voice, and to utter it under such circumstances that all the medical world is bound to hear.

The great American physician, as he well may, sighs for some, at any rate, of the old ways. He does not dispute the advance of knowledge, of knowledge of a certain kind. But he doubts the growth of practical capacity in the younger specialists; and he doubts it because he thinks that the "bedside instincts" are not cultivated at the present time. Not only are they not cultivated, they are despised. The specialist thinks much more of his specialty than he does of his patient. "In large cities," we are told, "the thorough, all-round, general practitioner is becoming scarce. . . . He is expected to be but the city directory, or the agent for the specialist in brains and nerves, in kidneys and appurtenances, in uterus and appendages, in skin and corns, in heart and lungs, in stomach, throat, nose, eyes, ears, and what not." All this the all-round man of wide knowledge and incomparable because incomparably trained "bedside instincts" like Dr. Jacobi, profoundly deplores. It is ruining medicine as an art. It is destroying the morale of general practitioners on an enormous scale. According to our venerable American critic, we shall very shortly have to look for the accomplished physician, in the old-fashioned and still proper sense of that term, not in large towns, but in outlying country villages, where it is impossible for the patient to insist upon a specialist for every trumpery ailment, and impossible also for the weak-minded and timid practitioner to yield against his better judgment to the patient's irrational and ignorant wish.

Specialism has now been predominant in the profession for a long time: for a time sufficiently long to enable us to make a reasonable estimate of its worth to medical science in general, and to the public who utilise medical science. Undoubtedly specialism has done good service, and is still doing good service in medicine. From the point of view of the increase of knowledge it is quite indispensable that the different parts and organs of the body shall be separately studied, and studied with such fullness of detail that every fact which can be discovered about them shall be discovered, and when discovered shall be fully made known. Moreover, different men have varying aptitudes and varying opportunities for the study of different parts and organs. Specialisation in the study of parts and organs is therefore clearly imperative if knowledge is to grow and intelligent comprehension is to become universal.

But let us turn for a moment from science to art, from knowledge to practice. We make bold to affirm that in practice, the man who is a "specialist" and not also a "generalist" is bound to be a complete failure. Not only is this the case with the specialist physician whose art consists mainly in the prescribing of drugs, it is the case also with the specialist surgeon, whose tools are the knife and the cautery. For both of these, and for the surgeon as much as the physician, a competent knowledge of general physiology, an intimate acquaintance with pathological processes and possibilities, a ready familiarity with the physiological actions of remedies, and an infinite variety of bedside therapeutic resources—all these are absolutely indispensable. One of the greatest, perhaps the greatest specialist of the present generation, is Mr. Jonathan Hutchinson, and his greatness as a specialist is absolutely and entirely the fruit of his greatness as a generalist. One of the most successful surgeons in Europe of pre-antiseptic days was the late Professor Spence, of Edinburgh, and his acknowledged pre-eminence at the bedside was, as is well-known, mainly due to his thorough knowledge of disease in its every variety and of all-round therapeutical resources.

Dr. Jacobi has not uttered his protest a moment too soon. If scientific medicine and a scientific therapeutic as a whole are to be preserved and to advance, the specialist must now be adjudicated upon; and what is called the "pure specialist" must be relegated to his proper level with dentists and other mongrel practitioners. In surgery, where delicate manipulation is required, as in operations on the eyes and the teeth, the pure specialist may still be tolerated, though even in these departments we look for the time when the general surgeon shall have the necessary skill, and the mere manufacture of teeth shall be relegated, as it now largely is, to the skilled mechanic. But in medicine, as distinguished from surgery, whilst different men will still be, so to speak, the patrons and expositors of different organs, the special practitioner will gradually cease to be. The old-fashioned general physician, specially instructed, specially trained, with all the variety of resource which patient study and many years of all-round experience and practice can alone command, must necessarily come to a speedy resurrection. The youthful specialist, whose knowledge is too often not even knowledge, but a mere farrago of opinions and crude speculations borrowed from the ephemeral literature of all nations, must as inevitably go under. How can it be otherwise? We have all found him out. We have all sent patients to him again and again, with almost invariably the same result, that whilst our patients have been in no wise advantaged, their pockets and our own have been lightened to an extent the reverse of agreeable. We doctors are often very slow to learn, but when we do learn we learn thoroughly; and we have learnt now by many a disappointing experience that the "pure specialist," as he proudly calls himself, is "a mockery, a delusion, and a snare." Having learnt this, we are coming back to the proper study of medicine as a whole; and it is quite certain that both medical science and the public advantage will be served by our change of front.

